# Supplementary figures and images for: Long read single cell RNA sequencing reveals the isoform diversity of Plasmodium vivax transcripts
Source: PLoS Negl Trop Dis. 2022 Dec 16;16(12):e0010991. doi: 10.1371/journal.pntd.0010991 (PMC9803293; doi:10.1371/journal.pntd.0010991)

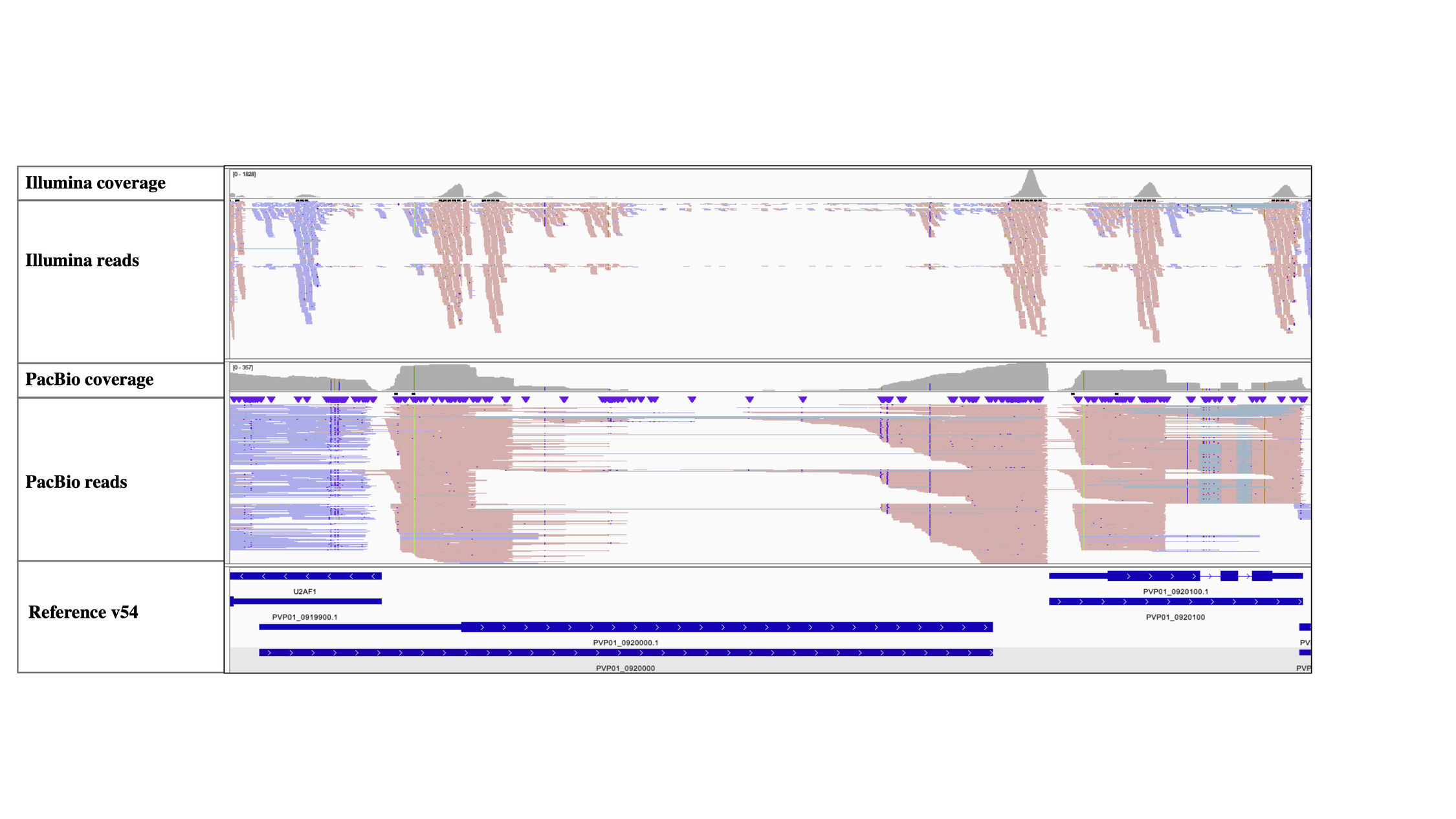

Supplement: S1 Fig — The top panel shows the data generated by Illumina sequencing and displays typical peaks corresponding to the 3’-end of each expressed transcript. The middle panel shows data generated, from the same mRNAs, using PacBio sequencing and illustrates how generating full-length transcripts improves interpretation of scRNA-seq data for organisms with incomplete gene annotations. (TIF) [file pntd.0010991.s001.tif]

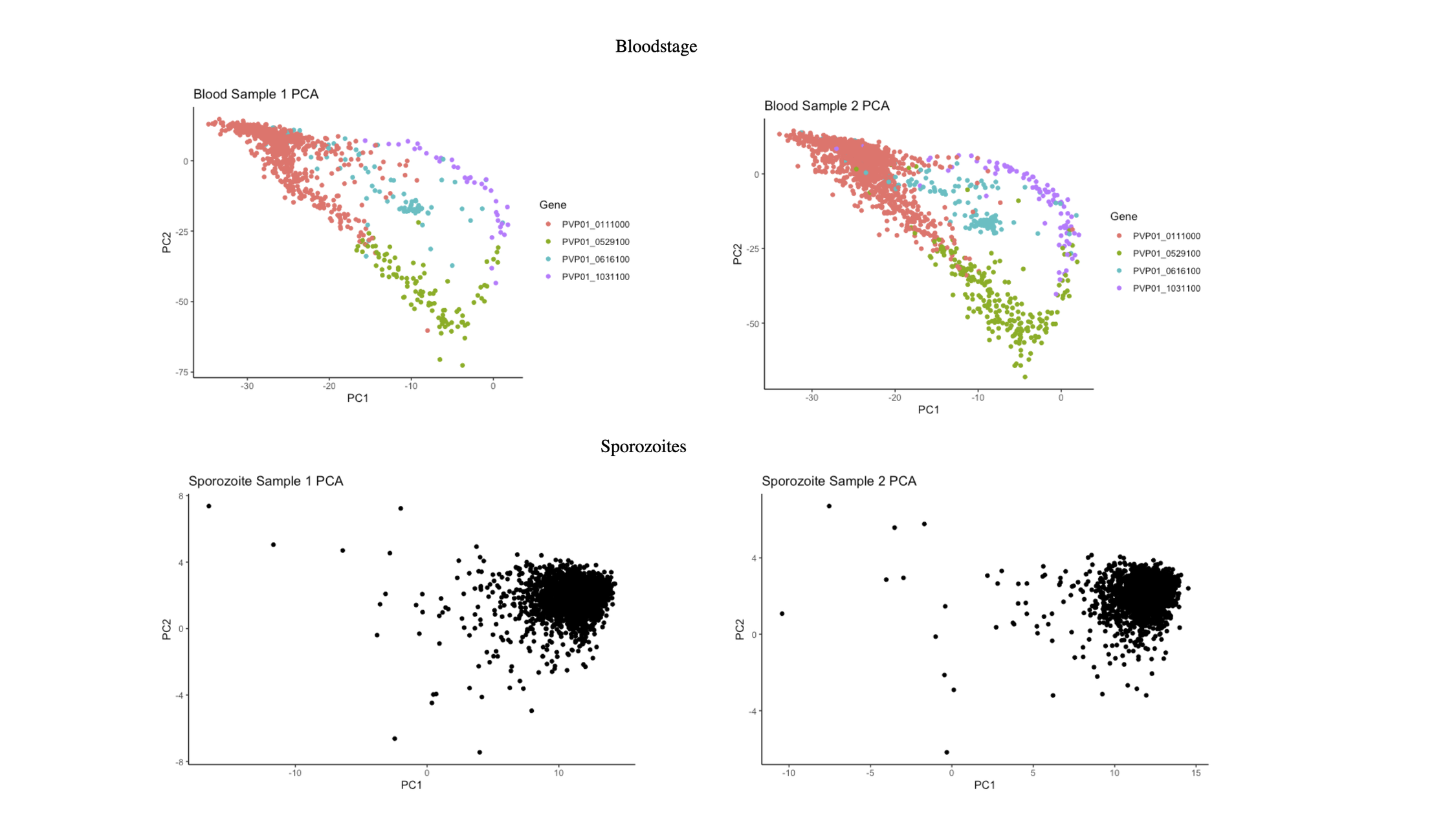

Supplement: S2 Fig — Top row: each dot is a single cell blood stage transcriptome, and is displayed based on its gene expression profile and colored according to the expression of stage markers (with cells being assigned to marker with the highest expression): red–early trophozoites, green–late trophozoites, purple–schizonts, turquoise–female gametocytes. Note that few ring stage parasites are included (if any) due to the enrichment method used (see Material and Methods). Bottom row: single cell sporozoite data. (TIF) [file pntd.0010991.s002.tif]

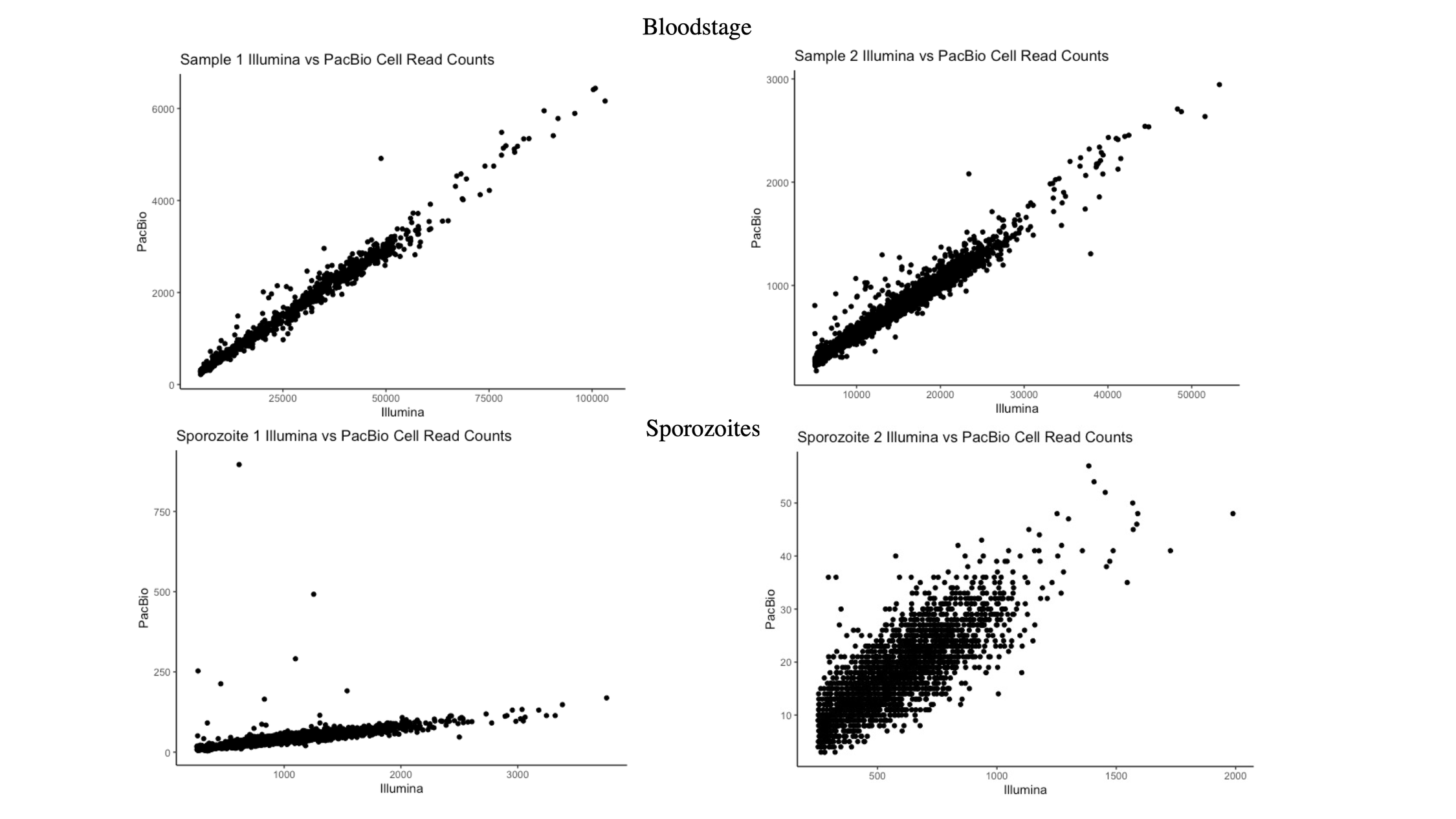

Supplement: S3 Fig — The scatterplot shows the correlation between the number of Illumina reads (x-axis) and PacBio reads (y-axis) obtained from each cell (individual black dots). Each panel represents the data for a different sample. (TIF) [file pntd.0010991.s003.tif]

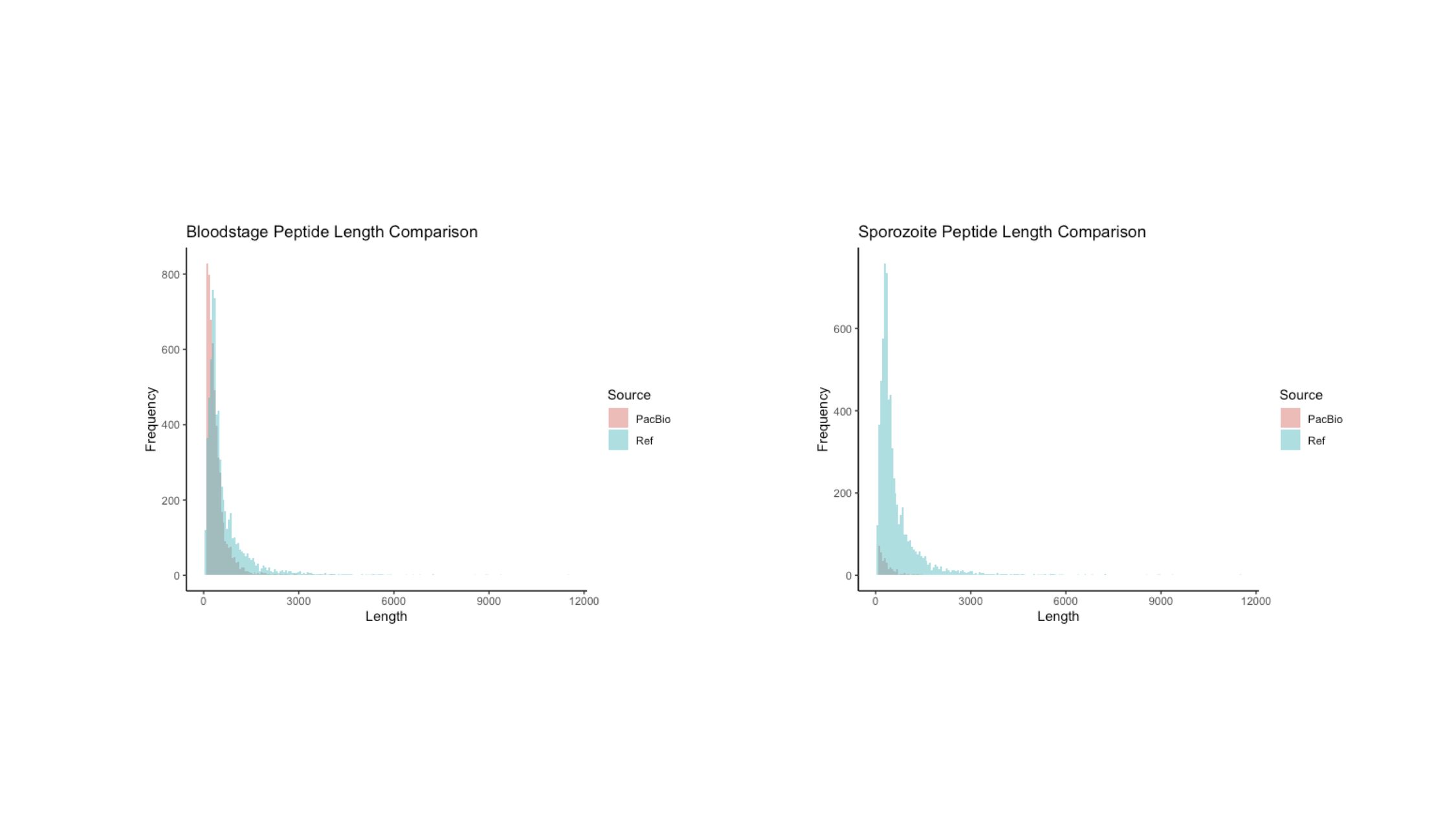

Supplement: S4 Fig — Distribution of the length (in amino acids) of the protein-coding sequences predicted from the PacBio transcripts (in red) and of the protein-coding sequences annotated in the P01 P. vivax genome (in blue). (TIF) [file pntd.0010991.s004.tif]

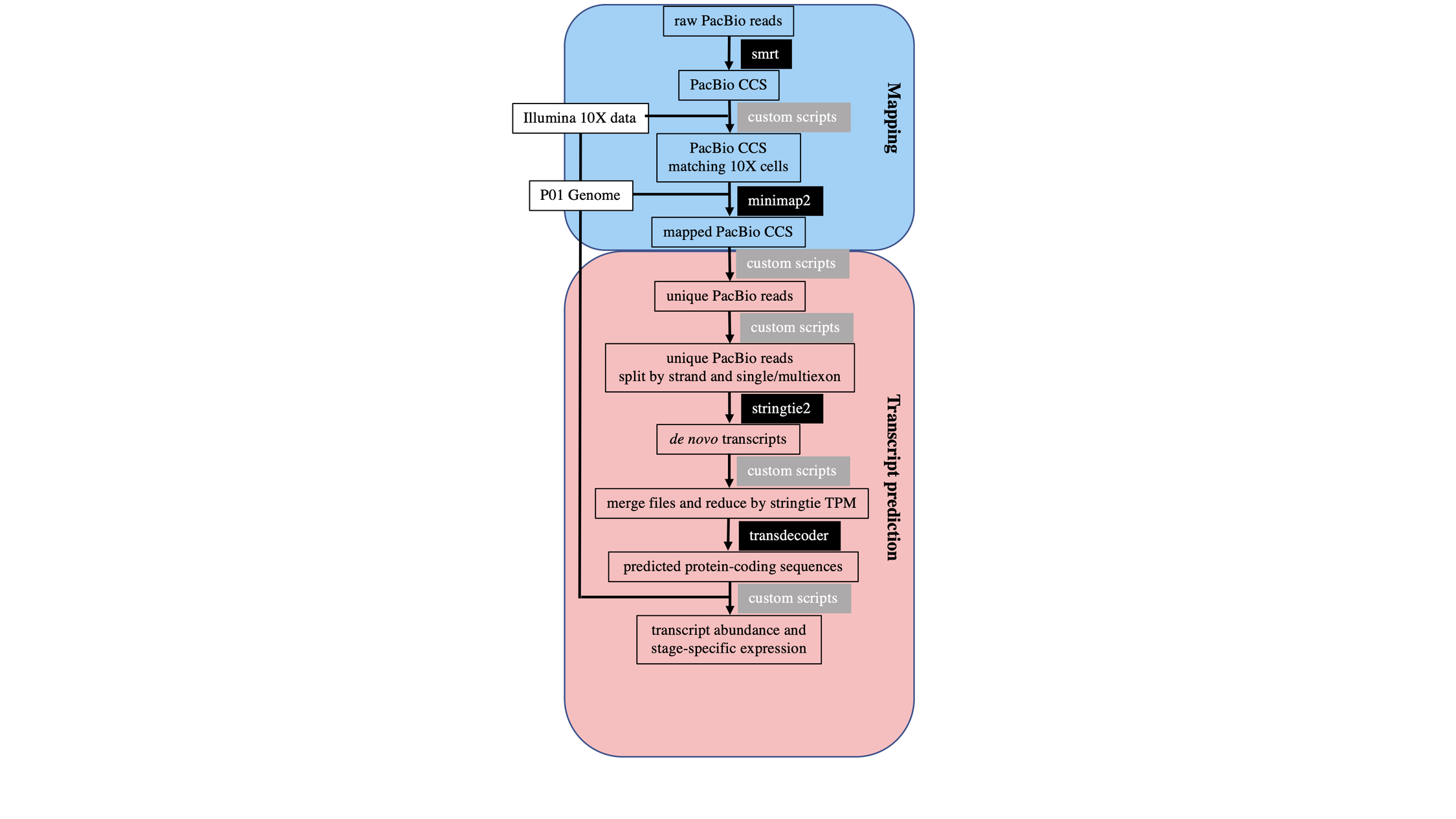

Supplement: S5 Fig — (TIF) [file pntd.0010991.s005.tif]

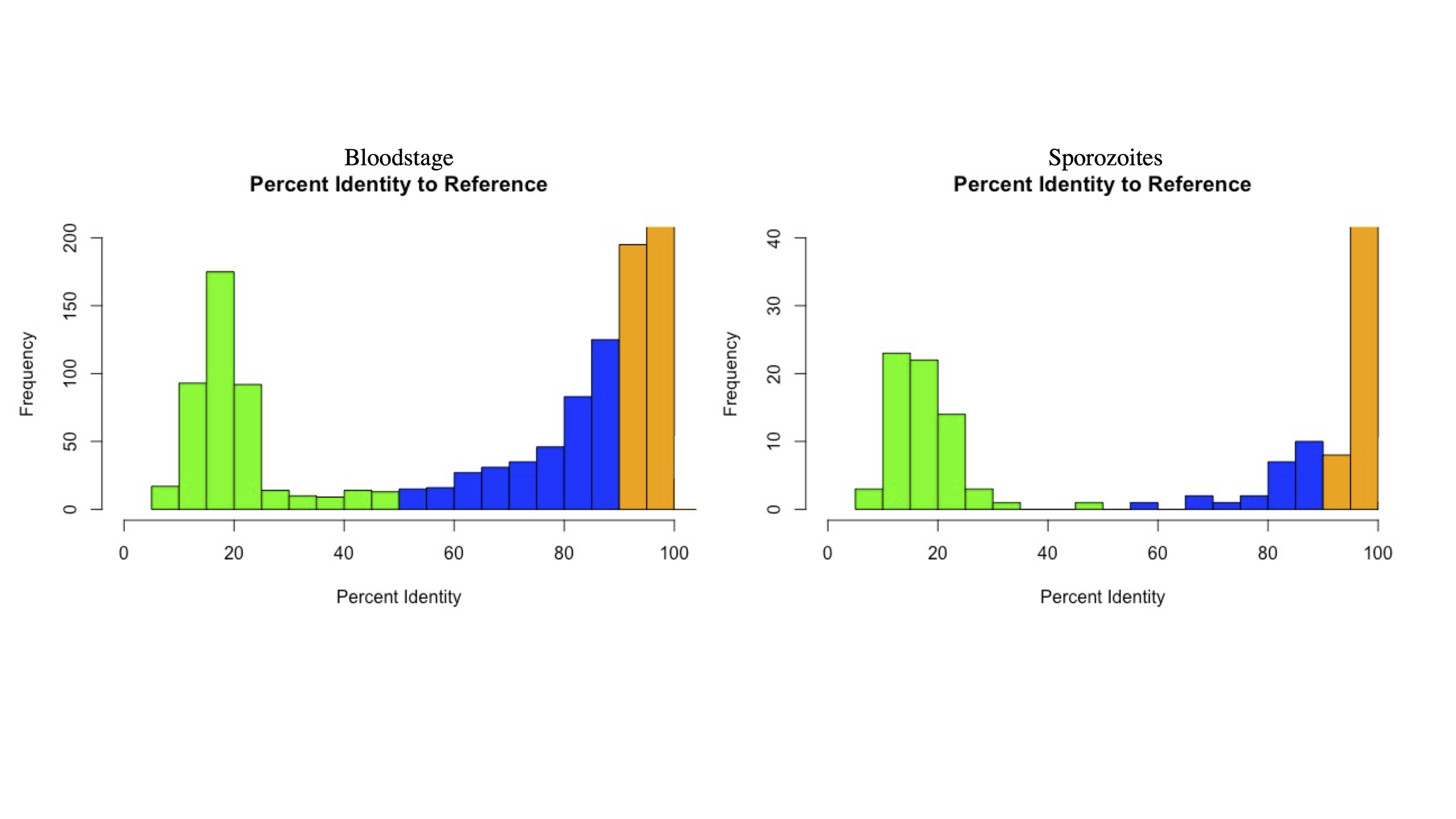

Supplement: S6 Fig — Distribution of the percentage alignment (x-axis) of the predicted protein coding sequences with the most similar protein sequence annotated in the P01 genome. Left: blood-stage transcripts. Right: sporozoite transcripts. (Note that the y-axis is cut and the right-most bars (perfect matches) go to 400 and 250 for the left and right panels, respectively). (TIF) [file pntd.0010991.s006.tif]

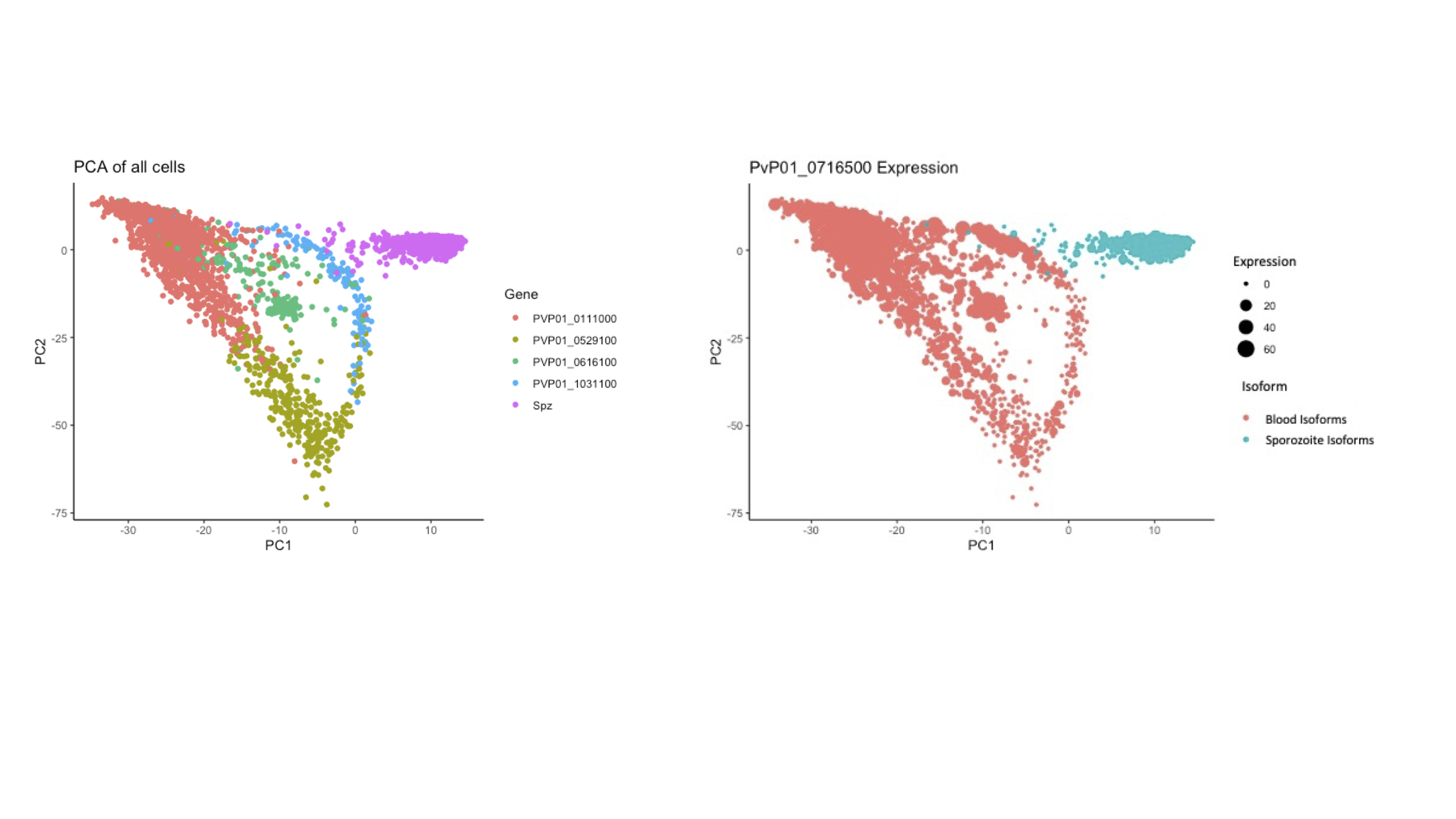

Supplement: S7 Fig — The left PCA shows blood-stage and sporozoite parasites jointly displayed according to their gene expression profiles. The right figure shows the same PCA, with each parasite colored based on the cytochrome b5-like heme/steroid binding protein isoform expression: red–“sporozoite” isoform, blue–“blood-stage” isoform. (TIF) [file pntd.0010991.s007.tif]

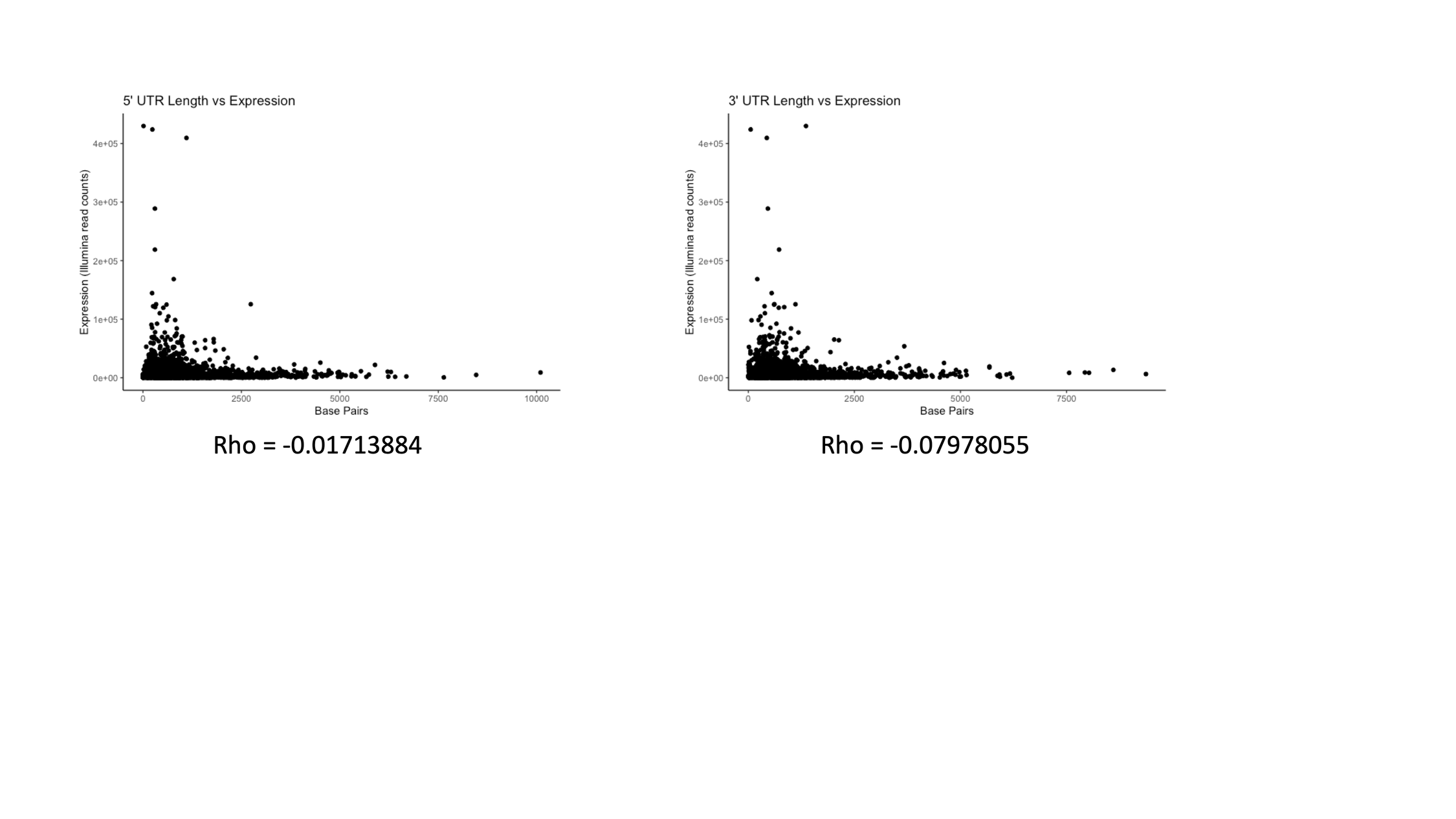

Supplement: S8 Fig — Correlation between the length of a transcript’s UTR (x-axis, in bp) and its level of expression determined by Illumina data (y-axis). Note that only genes expressing a single isoform are included in this analysis. (TIF) [file pntd.0010991.s008.tif]

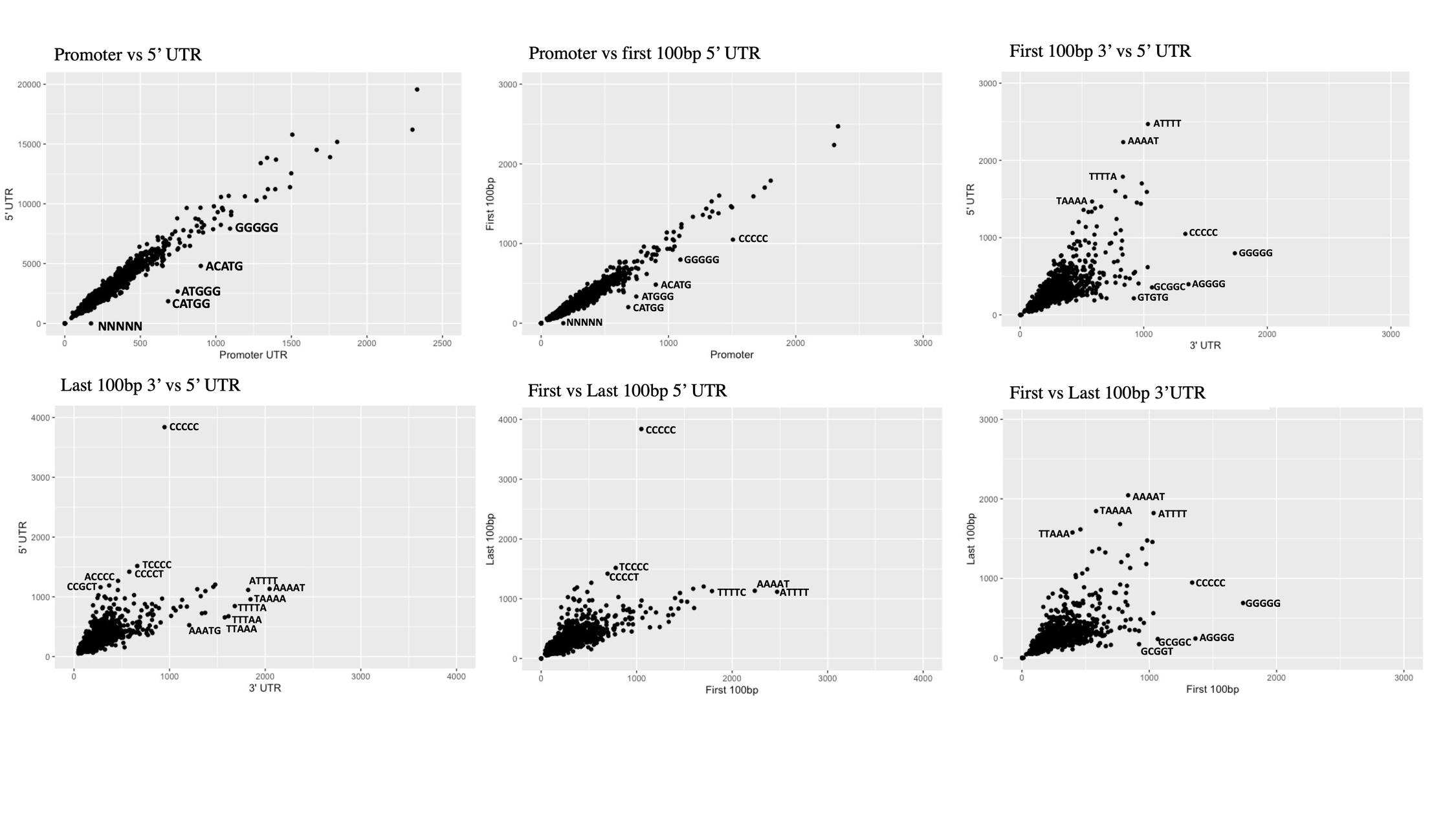

Supplement: S9 Fig — Comparison of the abundance of all 5-mers in gene promoters, 5’-UTRs and 3’-UTRs. Note that most motifs with different abundance (i.e., deviating from the diagonales) are either repeated sequences or encoding for a start codon (ATG). (TIF) [file pntd.0010991.s009.tif]

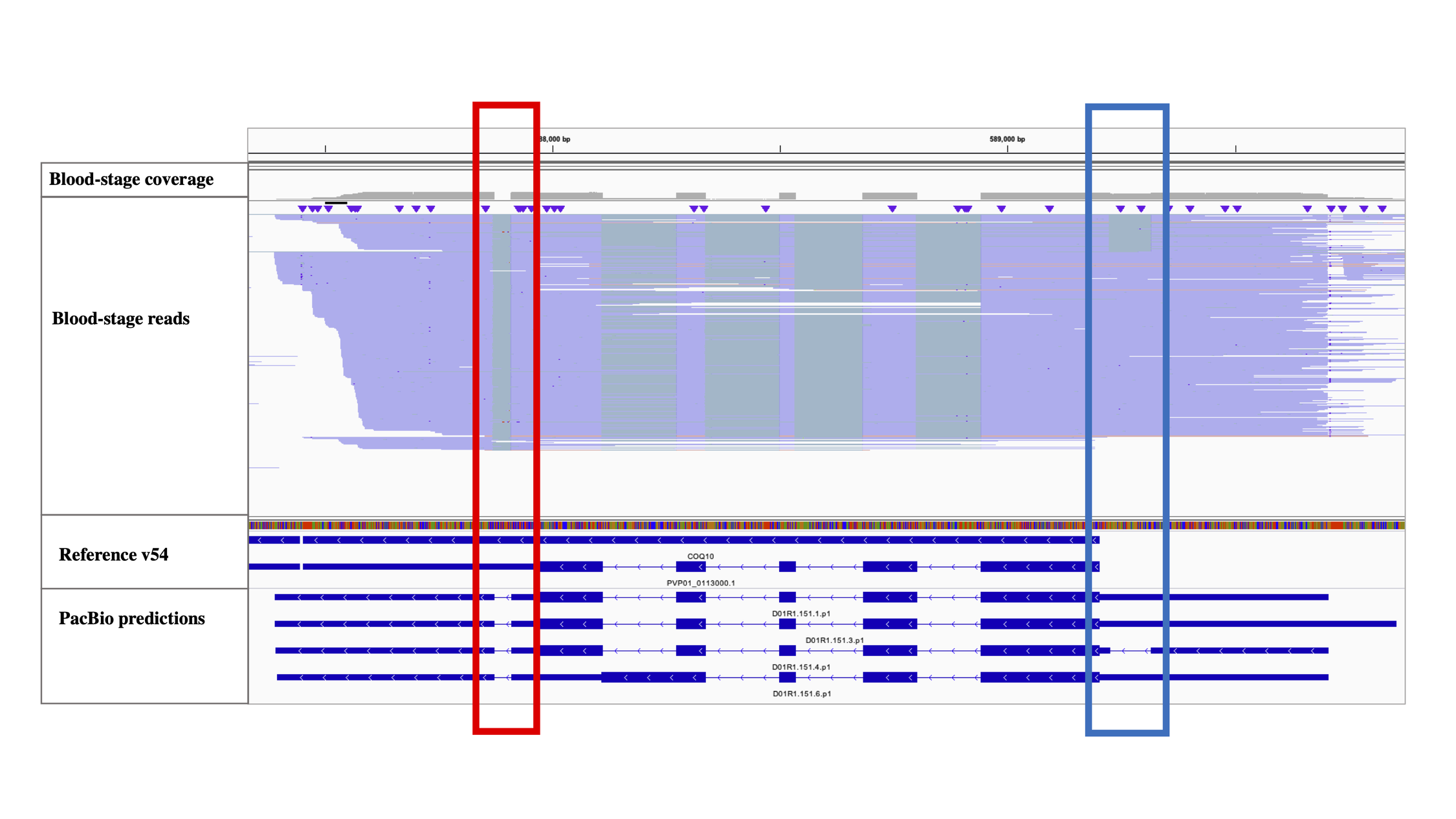

Supplement: S10 Fig — The figure shows PacBio reads (in blue) corresponding to the annotated mRNA for coenzyme Q-binding protein COQ10 homolog (PVP01_0113000) but with an unannotated intron in the 3’-UTR (red box) as well as, for a subset of the mRNAs, a second intron in the 5’-UTR (blue box). (TIF) [file pntd.0010991.s010.tif]

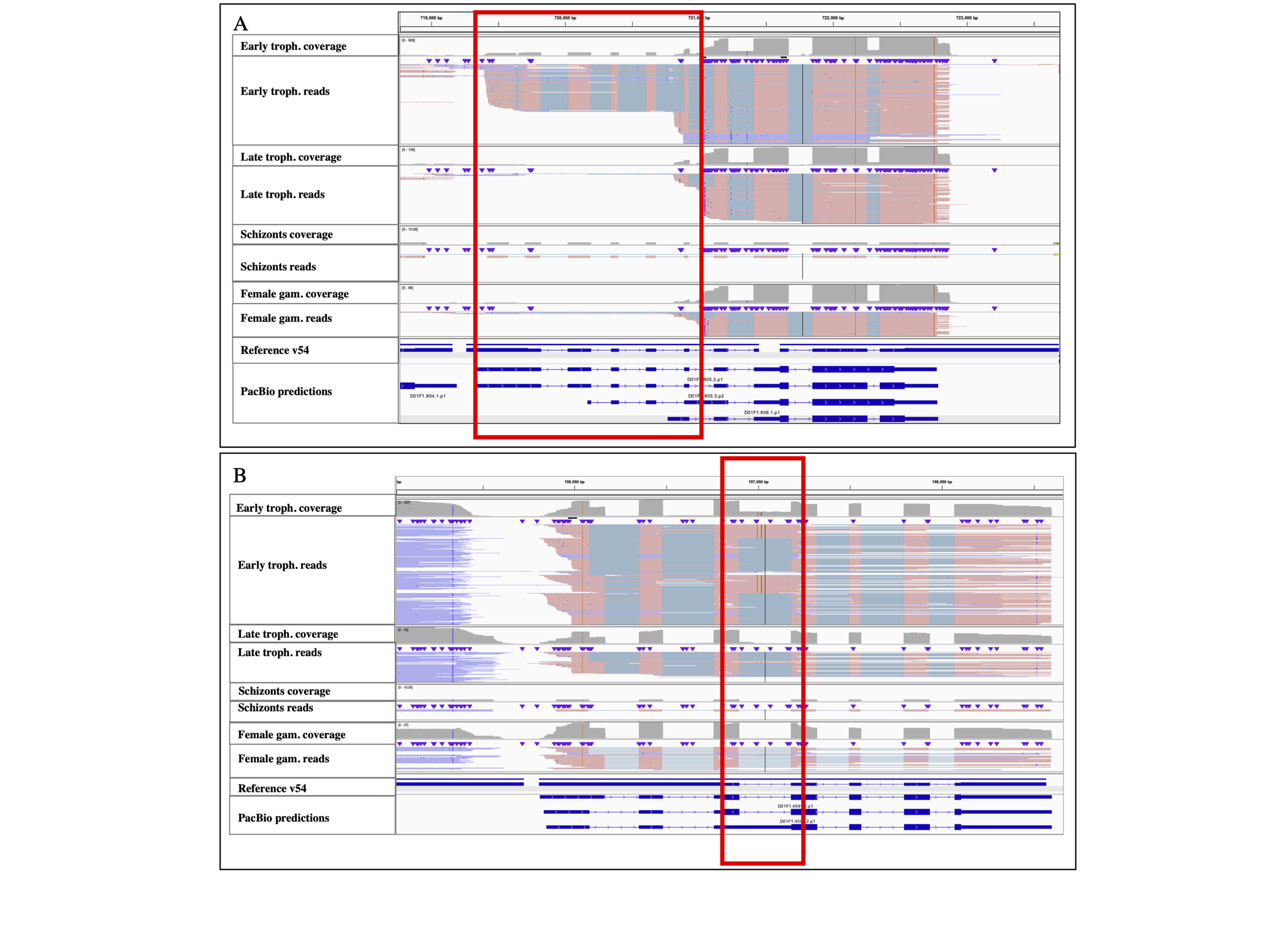

Supplement: S11 Fig — Each panel shows the PacBio reads mapped to a selected locus and split in four groups according to the stage of the parasites they derived from: early trophozoites, late trophozoites, schizonts and female gametocytes (from top to bottom). (A) Early trophozoites express the Ham 1-like protein (PVP01_0316500) from a more upstream TSS than the other stages and the resulting transcripts have a longer 5’-UTR containing five introns (red box). (B) The isoforms expressed from suppressor of kinetochore protein 1 (PVP01_1105000) result into different predicted protein coding sequences: some transcripts expressed exclusively in early trophozoites retain the third intron (red box) leading to a different open reading frame (blue bars at the bottom). (TIF) [file pntd.0010991.s011.tif]
