# Supplementary material for: Long read single cell RNA sequencing reveals the isoform diversity of Plasmodium vivax transcripts
Source: PLoS Negl Trop Dis. 2022 Dec 16;16(12):e0010991. doi: 10.1371/journal.pntd.0010991 (PMC9803293; doi:10.1371/journal.pntd.0010991)
Supplement: Attachment — Submitted filename: Response_PlosNTD_final.docx [file pntd.0010991.s017.docx]

We would like to thank the reviewers for their kind assessment of our manuscript and their constructive comments and suggestions. We have now modified the manuscript according to their suggestions and present below a detailed response to their comments (the page/line numbers refer to the manuscript without track changes).

**Reviewer #1**

Hazzard et al have generated short- and long-read scRNAseq data using the standard 10x genomics pipeline in combination with pacbio sequencing in P. vivax blood stages and sporozoites. Their analysis identifies a subset of transcripts that do not match the current reference annotations as well as isoforms that show a stage specific pattern of expression. They identify different UTR lengths between blood-stage parasites and sporozoites, and UTRs that contain introns, which they hypothesize might play a role in gene regulation.

Overall, I found the analysis superficial and lacking of interesting biology. Although this is an interesting dataset, the authors have not invested enough in the analysis to either reveal noteworthy biology or provide the community with a valuable resource dataset. I suggest strengthing the paper as a reference dataset prior to publication.

While we acknowledge the limitations of this study, we find the reviewer’s assessment of this work overly negative. This study is, as far as we know, the first attempt to comprehensively characterize variations in transcript isoforms between stages for a *Plasmodium* parasite. Our manuscript describes detailed laboratory and analytical procedures necessary to implement this analysis for any eukaryotic organism, and provides novel insights on the regulation of gene expression throughput the development of *P. vivax* parasites. In particular, the observation that different stages can express different isoforms of the same gene is novel and the extensive variations observed in UTR lengths and splicing was only preliminarily characterized before. We also now provide a gtf file (as suggested by the reviewer) to make this dataset more useful for the community.

Major Comments

The authors show that only half (or less) of the reads map within 500 bp of an annotated p. vivax gene and suggest that this is because of incomplete annotation of the reference genome. Can the authors use their data to improve the reference and demonstrate that more of the reads would map if they had better annotations?

Thank you for the suggestion. We have now implemented the suggested analyses and demonstrate that significantly more scRNA-seq reads map within annotated transcripts when the reference annotations are complemented with the PacBio predictions (lines 195-204, Supplemental Table S1). Briefly, while 27-33% of the scRNA-seq reads mapped within previously annotated genes for the blood stage parasites and 25-26% for the sporozoites, including the new PacBio predictions raises these figures to 69-77% and 65-70%, respectively. Since the predictions and the Illumina reads are based on the same molecules, we also evaluated the improvement in mapping using an independent scRNA-seq dataset (the AMRU-I blood stage parasites from Sa et al., 2020) and observed that using our PacBio predictions improved the mapping of these scRNA-seq reads in annotated transcripts from 30 to 88%.

line 196: "More in-depth analyses will be required to understand the function of these UTRs but the data presented here will provide a solid foundation to implement these studies." If the authors want this to be a useful dataset they need to provide the actual data. Could the authors provide a gtf with the newly annotated isoforms and UTRs? Can the dataset be integrated into PlasmoDB?

We thank the reviewer for this important suggestion. We unfortunately have no control on which data get added into PlasmoDB (or when) but have uploaded to SRA all files required to facilitate this process. We have also provided the data to the Ensembl annotation team. Finally, we now include a gft file (Supplemental information) containing all new isoforms as suggested by the reviewer.

Three of the five main figures are screen shots from a genome browser tool. I suggest that the authors diversify and include more information in the main figures. For example, where they demonstrate different isofroms between blood stage and sporozoite parasites, what does expression of the different isoforms look like on the PCA and does that help them interpret the biology of the cell clusters within the blood stages?

We have now moved some of the screenshots into Supplemental information (Fig S10 and Fig S11) and included PCAs displaying isoform expression as recommended by the reviewer (Fig 4B and Fig S7). Thank you for the suggestion.

The data collection methods are clear, although more background could be given to put the methods in context. The code used for the analysis is not available on the github link provided.

We have now added references to similar studies conducted in other organisms (lines 66-69). We apologize for the previously empty github link. All codes should now be available.

Minor Comments

-The authors should cite papers outside of malaria that have used this technology before, there is no context given for the development of the technology and what it has been used for in other systems.

Those studies are now referenced (page 4, References 30, 34, 35).

- The authors justify using pacbio to look at isoforms because the 10x libraries are 3' biased. However, they don't show that this is actually the case for P. vivax. Could the authors show this coverage bias in their data?

The 10X assay we used (3’end scRNA-seq) relies on sequencing mRNA molecules from their 3’ end after fragmentation. Based on this chemistry, only the last ~300 bp of each molecule are sequenced (regardless of the organism). We previously described this “bias” (more a characteristic of the assay) in our initial *P. vivax* scRNA-seq data (Sa et al., 2020) but this is now also shown on the new Supplemental Figure S1.

-Fig S1	Blood stages: show expression of markers so it is clear how stage was assigned.

Sporozoites: Colour the plot by a marker gene or UMI. Is there a subset of higher quality cells that could be used

We are not entirely sure to understand the reviewer’s request. We have now included the expression level of each marker gene on (now) Figure S2 (as well as on Figure S7). Please, note also that all the stage-specific analyses for asexual parasites rely exclusively on pseudotime, not classification using selected marker genes. For the sporozoites, we did not observe any meaningful variations among parasites (whether biological or technical) and they appear to form a relatively homogenous population.

-How have you controlled for different levels of expression in the stage-specific transcript analysis?

The stage-specific transcript analysis relies exclusively only absence/presence of the Pacbio transcripts in each cell, regardless of the expression level. We opted for this qualitative analysis due to the (relatively) low number of Pacbio reads which really hampers reliable determination of the read count per cell. This is now explicitly state on lines 352-356.

**Reviewer #2**

The submission by Hazzard and colleagues describes the application of combined Illumina/PacBio sequencing to 10X single cell transcriptomic analysis of Plasmodium vivax infected red blood cells. P. vivax remains a major cause of morbidity in large regions of the developing world, however analysis of these parasites has been hampered by the inability to raise this species in culture, thus limiting experimental approaches. The application of single cell transcriptomics to parasites isolated from patients or from experimentally infected primates has enabled a much better analysis of gene expression patterns, however technical limitations have resulted in most information being derived from sequences near the 3’ ends of the transcripts. Here the authors attempt to address this issue by using long-read sequencing technology, thereby deriving sequence information from much longer stretches of the transcripts, in many cases obtaining full-length transcript information.

This enabled them to identify products of alternative splicing and variations in the 5’ and 3’ UTRs. In addition, by identifying the developmental stage of each individual cell analyzed, they were able to associate stage-specificity with alternative isoforms, something that was difficult previously.

The manuscript reports an important and useful technical advance in the study of Plasmodium vivax. The methodology described here is likely to be applied by many research groups in the future, and the advance will likely also be applicable to other Plasmodium species, including P. falciparum. The manuscript also includes novel discoveries regarding alternative mRNA isoforms, which will undoubtedly be following up to determine their biological significance. Overall, the manuscript is well written and easily understood.

We thank the reviewer for the kind evaluation of our work and for highlighting how this approach could be useful for a wide range of researchers.

The conclusion section is remarkably brief and could be extended. The authors could mention the applicability of this methodology to other parasites, the possible significance of alternative mRNA isoforms and how this could be followed up and how the findings of this study improve our understanding of P. vivax biology.

Thank you for the detailed suggestions. We have now significantly extended the conclusion and included the topics raised by the reviewer (lines 270-284).

I have only minor comments for the authors to consider.

1. On line 147, the authors state that the length of the transcripts from asexual stages ranged from 100 to 20,506 bp. A 20 kb transcript is remarkably long, much longer than any annotated gene. What is this transcript and do the authors think this is a genuine mRNA?

The stringtie predictions occasionally lead to artefacts, when transcripts in the same orientation overlap, or if there are systematic mis-mapping. The 20 kb transcript seems to be a fusion of several overlapping transcripts (**Figure A**). This is now clearly stated on lines 150-152. Thank you for noticing this issue.

**Figure A.** Screenshot showing that the 20 kb transcript predicted by Stringtie (D01R1.3386.1.p1) is likely an artefact due to overlapping transcripts in the same orientation (PVP01_09021900, PVP01_09021800, PVP01_0921700).

2. In Figures 2C, 5A and 5C there appears to be evidence for alternative splicing that is not mentioned in the Figure legend. Is this correct? If so, it might be worth mentioning so that readers are not confused.

The reviewer is correct. We now mentioned these additional splicing products in the legend of these figures.

3. In Figure 5, the authors mark the figures with the terms “Group A”, “Group B” and “Group C”, while the figure legend explains that these terms refer to early trophozoites, late trophozoites and schizonts respectively. Why not simply mark the figure itself with the stage of the parasites? I don’t see the utility of using the terms “Group A, B, C”.

We have now included these terms on the figure (with a cautionary note).

**Reviewer #3**

The manuscript by Hazzard and colleagues presents a single-cell sequencing dataset consisting of Plasmodium vivax blood stages and sporozoites, sequenced using two different platforms – traditional Illumina sequencing and PacBio long-read sequencing.

Plasmodium vivax remains a relevant human pathogen and given how few good quality transcriptomics datasets are available, the presented work is certainly of significant value to the parasitology community. Additionally, the combination of the two techniques provides a wealth of information regarding the parasite transcript structure, gene expression changes etc. The rationale of the work is clearly stated and backed up by relevant literature. The manuscript is generally clearly written.

Thank you for the kind assessment of the work.

There is however a number of details (mostly related to data analysis) that should be clarified in order to justify some of the conclusions of the manuscript. The major ones are:

1) Cell identity:

It is not clear how different stages of the parasite life cycle were identified in the blood sample. The single-cell data appears to be plotted as PCA components (rather than UMAP or t-SNE graphs which are the golden standard of dimensionality reduction for this data type) and does not form clear clusters. There is no mention of any attempts of clustering, identification of marker genes or developmental trajectory mapping etc. Pseudotime analysis is mentioned but there are no methods attached to it, so it is unknown what was done exactly.

We used PCA, rather than UMAP or tSNE, primarily for its ability to better recapitulate continuous variations in gene expression: distances between points on a UMAP and tSNE representation are non-linear and these algorithms are designed to identify distinct groups of cells (e.g., different cell types). *Plasmodium* parasites in the blood are typically present at different “stages” of their intraerythrocytic development but these variations are continuous rather than discrete (with the possible exception of the gametocytes). For this reason, we (Sa et al., 2020, Bogale et al, 2021) and others (e.g., Reid et al., 2018, Howick et al., 2019) have previously chosen to use PCA to display relationships among the transcriptomes of single *Plasmodium* parasites throughout their development. This is also the rationale for using pseudotime analysis for analyzing variations among blood-stage parasites. We only use the microscopy terminology (e.g., ring-stage or schizonts) for sake of simplicity to describe parasites at different points of their development but caution the reader about the boundaries of these terminologies and the exactitude of the correspondence to microscopy-defined parasites (see e.g., legend of Fig 4).

The sporozoite samples are processed separately so it is not clear how they relate to other stages. Given the importance for further transcript assignment, the details of this analysis (if done) should be included, ideally pooling the sporozoite samples together with other stages.

We (Bogale et al, 2021) and others (Howick et al. 2019, Real et al., 2021, Witmer et al, 2021) have shown, using scRNA-seq data, that sporozoites differ dramatically from blood-stage parasites in their gene expression profiles and we therefore analyzed them separately. In addition, sporozoite scRNA-seq data suffer from host contamination, which dramatically reduces the number of reads available for characterizing single cell transcriptomes, and the cutoffs used for analyses need therefore to be adjusted accordingly (discussed on lines 108-111). To address the reviewer’s concern, we have also included a new supplemental figure (S7) showing sporozoites and blood-stage parasites together.

2) Full transcript annotation:

Authors mention themselves that numerous transcripts/peptides predicted from their annotation appear to be truncated blaming it on “the 10X reverse transcriptase processivity that will hamper synthesis of long cDNA molecules and prevent recovery of full-length transcripts”. In this light, the fact that the vast majority of the new isoforms they identified differ in the length of 3’UTR or 5’UTR only, may suggest that they represent truncated RNA molecules rather than full functional transcript isoforms. The fact that UTRs in general have different lengths between the stages (something not observed in the other Plasmodium datasets as far as I know) may support this.

In this case more stringent filtering conditions (or an attempt to validate at least one of the "new' transcripts by eg. Northern blot, although I realise that authors may not have sufficient material or the tools to attempt it) need to be used to identify the novel Plasmodium isoforms This should be acknowledged in the manuscript and the general conclusions regarding the transcript UTRs length should be toned down.

The reviewer raises an interesting point but we do not think that the variations in UTRs observed are caused by analyses of truncated RNA molecules resulting from the lower 10X RT processivity. First, all transcripts considered in our analyses are supported by at least 10 independent reads (i.e., derived from >10 distinct mRNA molecules) in two samples, which should filter out most partially reverse-transcribed molecules that are unlikely to be truncated at exactly the same position. Second, we would expect reduced processivity to have little-to-no impact on the 3’-UTRs since the polyA tail serves as priming for the RT and, in our data, we observed similar patterns in 3’- and 5’-UTRs (see e.g., Figure 3 and Table 2). Third, many of the UTR length variations described are caused by the presence of introns in the UTR rather than variations in UTR termination. Fourth, we did not observe any correlation between gene length and UTR length (see Figure B below) while, if RT processivity was a major confounder of UTR length, we would expect longer genes to have shorter UTR. As the reviewer notes, validating these transcripts by northern blot is unfortunately unfeasible.

|  |  |
| --- | --- |
| **Figure B.** Scatterplots showing the predicted lengths of the 5’-UTR (left) and 3’-UTR (right) relative to the predicted protein coding sequence length (x-axis). | |

3) Differential isoform usage between the life stages.

Again, it is not clear how robust this analysis is without further details. Rather than using tools for isoform usage quantification, the authors choose to go for custom approach consisting of the stage distribution of cells containing a given isoform across the life cycle (if I understand correctly). No details of this method and no processed output is given. The manuscripts is badly missing a table with the list of all stage-specific isoforms, the numbers of molecules that their analysis is based on, and the results of statistical test applied to each of them. More details (or validation of one of the genes with >1 isoforms) are required to substantiate the authors claim regarding the stage-specificity.

We wanted to specifically test whether different isoforms of the same gene were used at different times of the parasite development and, since the relatively low read depth of the PacBio prevents accurate quantitative estimates of expression, we developed this custom qualitative analysis. For each gene that had two (or more) isoforms, each expressed in >50 cells, we compared the pseudotime ranks of the cells expressing the first isoform with the pseudotime ranks of the cells expressing the second isoform using a Kolmogorov-Smirnov test (this is not clearly presented on lines 352-356). We have now added a supplemental table (Table S4) with the list of all stage-specific isoforms and the results of the statistical testing. Thank you for this suggestion.

Additionally:

1) there are no attempts to compare the author’s dataset to similar ones generated for P.falciparum, a model human malaria species. As the two parasites share very similar syntenic genomes (with 1:1 orthologs for almost all genes) and similar exon/intron structure, such comparison could be very useful, given the fact that P.f has much better UTR annotation and a number of stage specific gene expression studies

We are not entirely sure we understand the reviewer’s suggestion. Comparison of scRNA-seq data derived from different *Plasmodium* species has been conducted before (e.g., Howick et al, 2019 or Sa et al., 2020) and is beyond the scope of this study. Comparison of stage-specific isoforms between *P. vivax* and *P. falciparum* might be interesting but we do not have access to a similar dataset as described here for *P. falciparum*.

2) The authors don’t provide the link to raw datasets (eg. SRA, GEO).Given the declaration in the data availability statement, I assume that this is a last-minute omission and the data will be made accessible to the community

The SRA/Bioproject accession numbers have been updated (page 17) and released.

3) A Github repository with the scripts for the data analysis is empty.

Our apologies for the empty link. The github page (page 17) should now be accessible.

4) it is not clear what the fig S7 should represent as the labelling of the axes is not very helpful. No statistical test is applied, and no raw numbers are given, so it is difficult to tell how significant the difference in kmers is. For the motif enrichment in the specific sequences, a number of standard tools are available (Meme, Ace etc.)

We have now modified the legend of Fig S7 (now S9) to highlight that most motifs with different abundance are either repeated sequences or encoding for a start codon.

4. In asexual stage annotation authors identify the trophozoites, schizonts and females but not the rings and males which should be also present in the mixed blood stages. Was that an artefact of the experimental design? Can authors provide reasons for that?

Thank you for pointing this out. We have now edited the manuscript (line 298-299 and Figure S2 legend) to clarify these points. Briefly, rings are largely missing from our dataset as a result of the enrichment method used for capturing infected red blood cells, while male gametocytes were excluded from the analysis as there were too few cells characterized (male gametocytes typically represent <1% of all blood-stage *P. vivax* parasites).

5. Based in the few examples shown in Fig 5, the transcripts with multiple isoforms tend to be more expressed at the stage in which a new isoform is identified. Given how many genes are differentially expressed between the life stages, it would be useful to include the results of differential expression analysis of the genes which are differentially spliced in order to see if one phenomenon I connected with the other (eg. is the increase of expression correlated with different UTR). That could also provide a hint whether some of the isoforms are just result of faulty transcript processing that appear whether the gene is highly expressed.

This is a very interesting hypothesis that we will need to explore further. However, we do not think that the data we have support faulty processing of highly expressed transcripts, despite what was (poorly?) represented previously on Fig 5: the identification of stage-specific isoforms was conducted at the single cell level (see our response above) and, in the majority of the cases, only one of the two isoforms was detected in each cell. Even when more than one isoform was detected per cell, the “minor” isoform was the most expressed in many cells (see e.g., new Figure 4B). It is therefore unlikely that the minor isoform represents faulty transcripts from highly expressed genes since it is often the most expressed (or only) transcript from that gene in a given cell. The new figures (Fig 4 and Fig S7) illustrate now that different isoforms are expressed in different cells.

Regardless of these omissions, the study is well designed, and its publication will be of interest for the malaria community. I commend the authors for their effort and looking forward to seeing this work in print.
